# Author Correction: Elucidation of the structural basis for ligand binding and translocation in conserved insect odorant receptor co-receptors

**DOI:** 10.1038/s41467-024-48896-9

**Published:** 2024-05-23

**Authors:** Jody Pacalon, Guillaume Audic, Justine Magnat, Manon Philip, Jérôme Golebiowski, Christophe J. Moreau, Jérémie Topin

**Affiliations:** 1 Université Côte d’Azur, Institut de Chimie de Nice UMR7272, CNRS, Nice, France; 2 Univ. Grenoble Alpes, CNRS, CEA, IBS, Grenoble, France; 3 Department of Brain & Cognitive Sciences, DGIST, 333, Techno JungAng, Daero, HyeongPoong Myeon, Daegu, Republic of Korea

**Keywords:** Computational chemistry, Patch clamp, Ligand-gated ion channels, Molecular dynamics, Bioinformatics

Correction to: *Nature Communications* 10.1038/s41467-023-44058-5, published online 11 December 2023

The original version of this Article contained an error in the ‘Western Blots’ section of the ‘Methods’ which incorrectly read ‘The polyclonal primary antibody anti-Orco was purchased from Genscript and designed against the peptide sequence SSIPVEIPRLPIKSFYPW in the second extracellular loop (ECL2).’. The correct version replaces this sentence with ‘The polyclonal primary antibody anti-Orco was purchased from Genscript and designed against the peptide sequence CYSCHWYDGSEEAKT in the fourth intracellular loop (ICL4).’.

This has been corrected in both the PDF and HTML versions of the Article.

The original version of the Reporting Summary associated with this Article included an incorrect ‘Antibodies’ section, in which the incorrect antibody information was provided. The HTML has been updated to include a corrected version of the Reporting Summary.

## Reporting summary

Further information on research design is available in the Nature Portfolio Reporting Summary linked to this article.

### Supplementary information

Updated Reporting Summary

