# Supplementary material for: Elucidation of the structural basis for ligand binding and translocation in conserved insect odorant receptor co-receptors
Source: Nat Commun. 2023 Dec 11;14:8182. doi: 10.1038/s41467-023-44058-5 (PMC10713630; doi:10.1038/s41467-023-44058-5)

Supplementary Information for

Elucidation of the structural basis for ligand binding and translocation in conserved insect odorant receptor co-receptors

Jody Pacalon[a,1], Guillaume Audic[b,1], Justine Magnat[b], Manon Philip[b], Jérôme Golebiowski[c], Christophe J. Moreau[b,*], Jérémie Topin[a,*]

[a] Université Côte d'Azur, Institut de Chimie de Nice UMR7272, CNRS, France

[b] Univ. Grenoble Alpes, CNRS, CEA, IBS, F-38000 Grenoble, France

[c] Department of Brain & Cognitive Sciences, DGIST, 333, Techno JungAng, Daero, HyeongPoong Myeon, Daegu, 711-873, Republic of Korea

* Jérémie Topin, 28 avenue Valrose, 06108 Nice France, +33 (0)4 89 15 01 32, jeremie.topin@univ-cotedazur.fr; Christophe Moreau, 71, avenue des Martyrs CS10090 F-38044 Grenoble cedex 9 France, +33 457 428 579, Christophe.moreau@ibs.fr.

This PDF file includes:

Supplementary Fig.1 to 11

Supplementary Tables 1 to 3

Legends for Datasets S1to S2

Supplementary References

Other supplementary materials for this manuscript include the following:

Datasets S1

Datasets S2

[1] These authors contributed equally to this work.

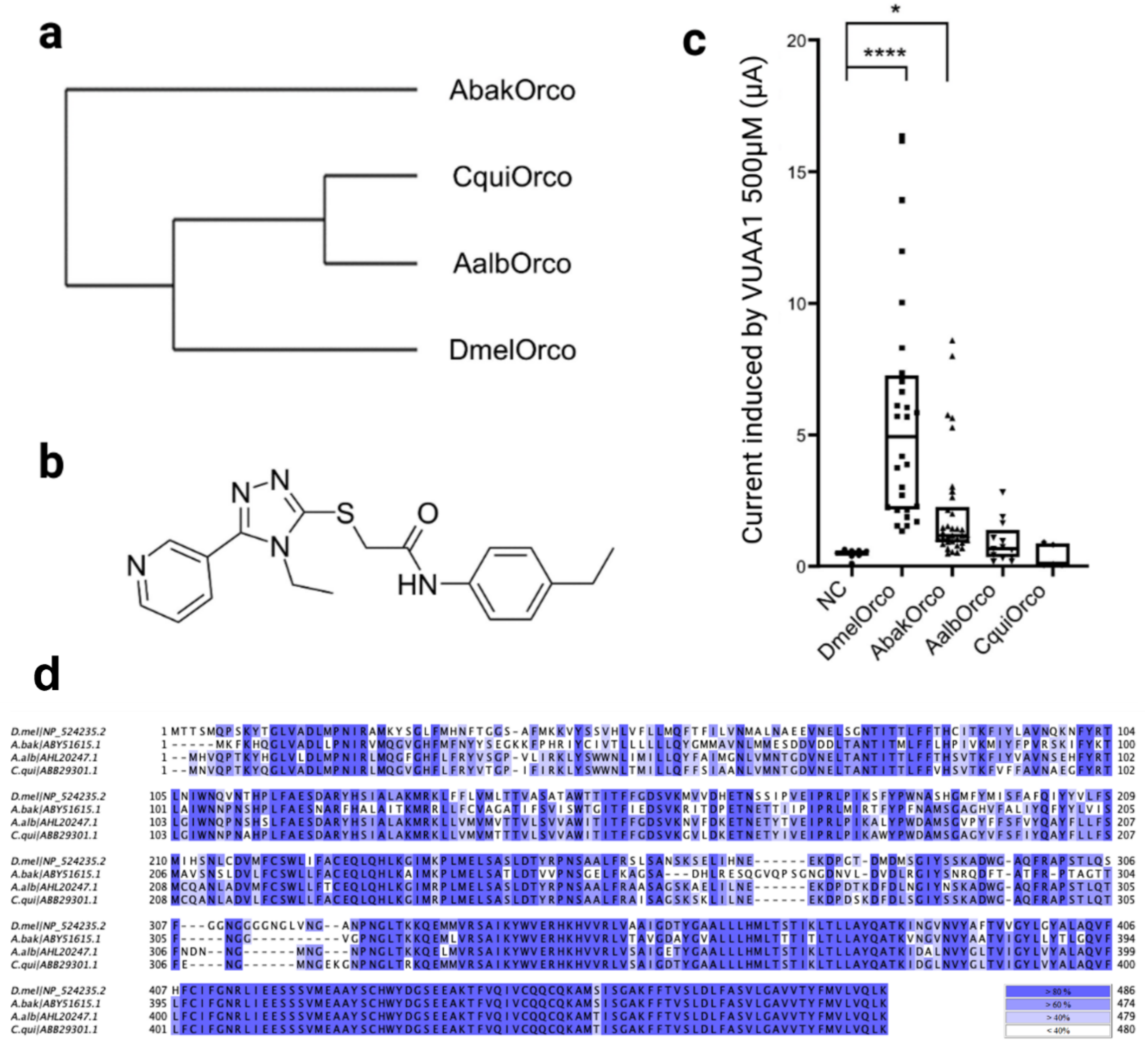

Supplementary Fig. 1 | Determination of the Orco species generating the highest response to VUAA1 in TEVC recordings. (a) Phylogenetic tree of Orcos from different species. Abak: *Apocrypta bakeri*; Cqui: *Culex quinquefasciatus,* Aalb: *Aedes albopictus*, Dmel: *Drosophila melanogaster*. **(b)** Structure of VUAA1. **(c)** Current amplitudes induced by 500 µM VUAA1 on Orcos from different species expressed in *Xenopus* oocytes and recorded by TEVC. Results are median +/- SEM. P values are <0.0001 (Dmel, ****), 0.0438 (Abak, *) and 0.92 (Aalb), with NC as reference in the Kruskal-Wallis test. n= 8 Negative control, n= 28 DmelOrco, n= 34 AbakOrco, n=13 AalbOrco, n= 5 CquiOrco; NC: negative control (water-injected oocytes). (**d)** Alignment of the 4 Orco species tested in this study. The amino acids are colored according to the percentage of the residues in each column that agree with the consensus sequence. Only the residues that agree with the consensus residue for each column are colored.

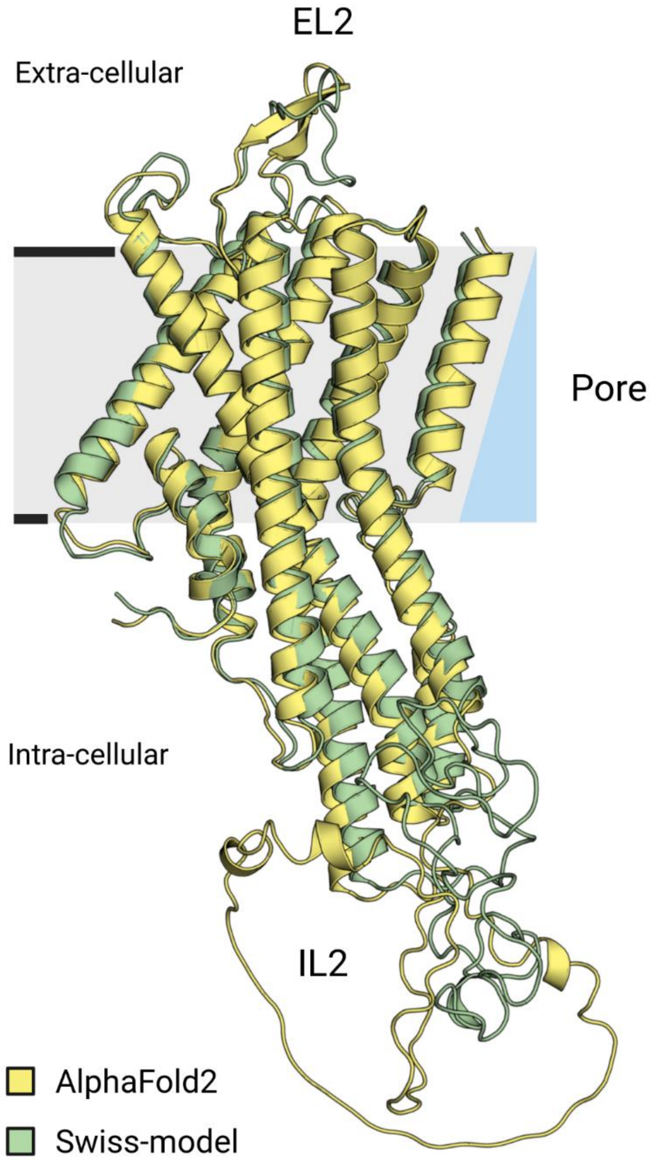

Supplementary Fig. 2 | Superposition of the AlphaFold2 DmelOrco model with the AbakOrco structure. Superposition of the experimental structure of AbakOrco homotetramer (pdb ID: 6C70) superposed on the AlphaFold2 model retrieved from the Alpha Fold Protein database (in yellow). The two structures have a RMSD of 0.7 Å calculated on their transmembrane segments. To orient the protomer, part of the pore is shown on right as blue right-angled triangle. EL2: extracellular loop 2. IL2: intracellular loop 2.

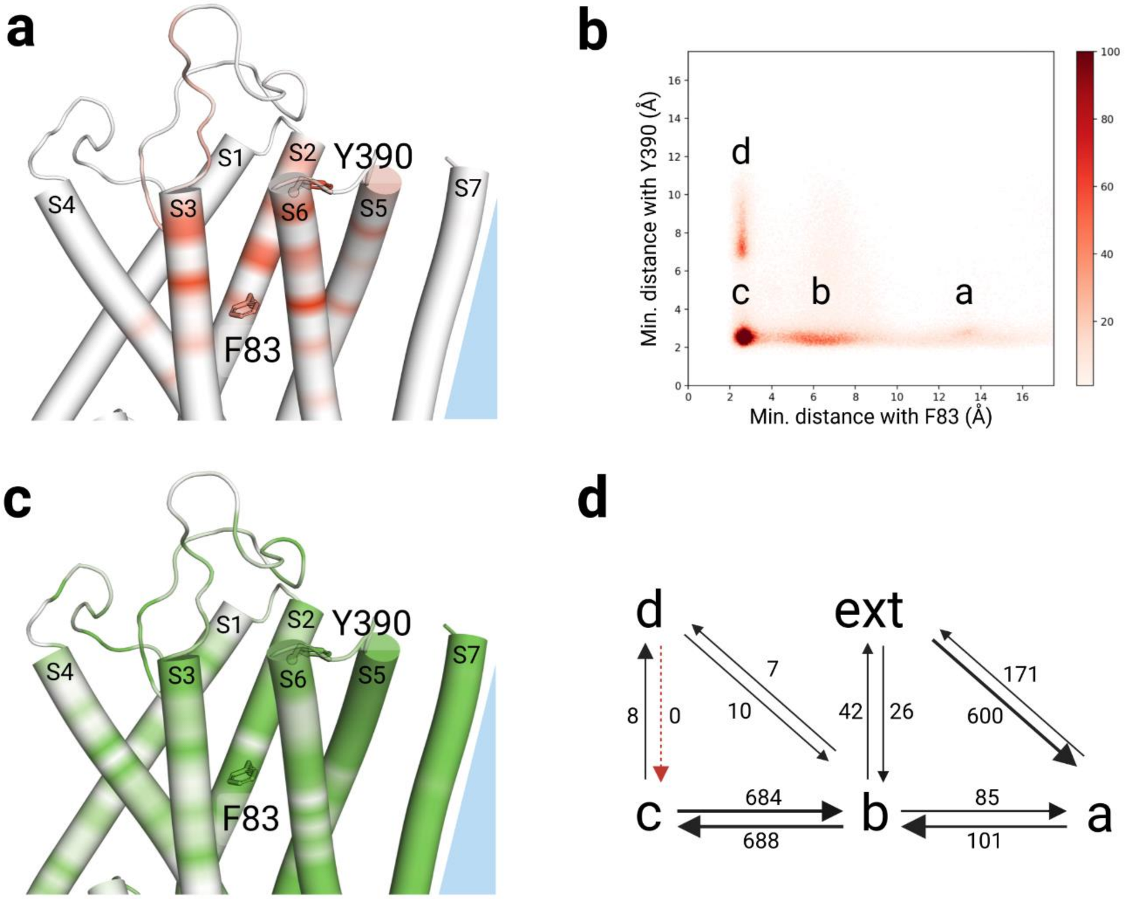

Supplementary Fig. 3 | Summary of the interactions observed during the MD simulations between VUAA1 and DmelOrco. (a) Contact frequencies of VUAA1 mapped onto the structure of DmelOrco. The color gradient account for high frequency interaction (in red) to no interaction (in white). **(b)** Contour map of VUAA1 migration as the minimum distance from $F83^{S2}$ (distance from the entry) and minimum distance from $Y390^{S6}$ (distance from the cradle of the cavity). The four basin allow identifying four states in the VUAA1 binding: contact a, entry b, vestibule c and binding d. **(c)** Residue conservation among 176 Orcos from 174 species mapped onto the structure of DmelOrco using a colour scale from green high conservation, to white low conservation. **(d)** Proposed mechanism of VUAA1 binding. The numbers indicate the transition from one state to another. ext: extracellular.

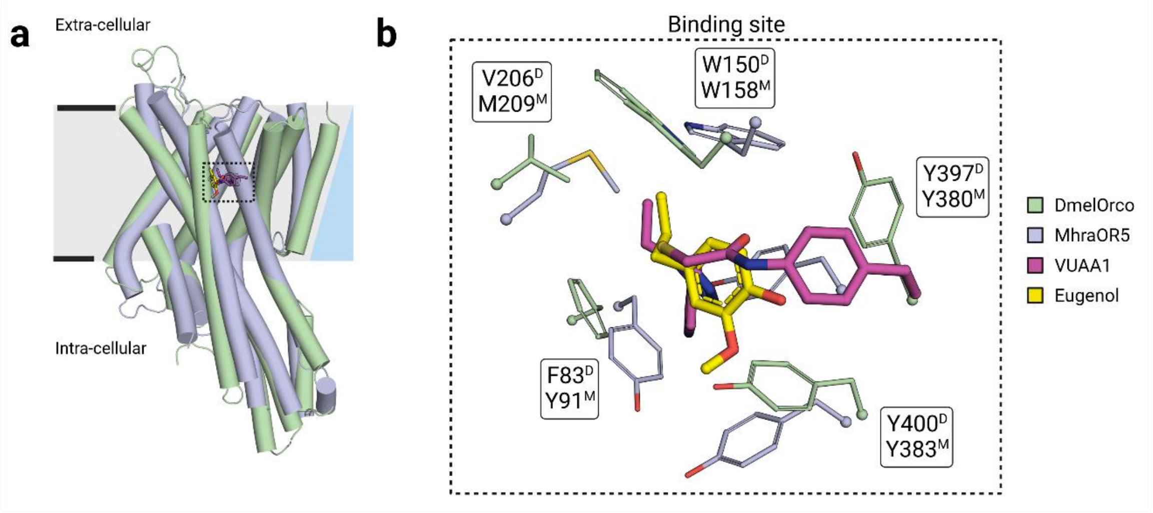

Supplementary Fig. 4 | Comparison of the binding site location predicted in DmelOrco and observed in MhraOR5 structure. (a) Superposition of DmelOrco (green tubes) and MhraOR5 (blue tubes), with their respective ligands VUAA1 (magenta sticks) and eugenol (yellow sticks). **(b)** Close-up view of the binding site of DmelOrco (green licorice) and MhraOR5 (blue licorice) with their respective ligands VUAA1 (magenta sticks) and eugenol (yellow sticks). The superscripts D or M on the amino acids names represent DmelOrco and MhraOR5, respectively.

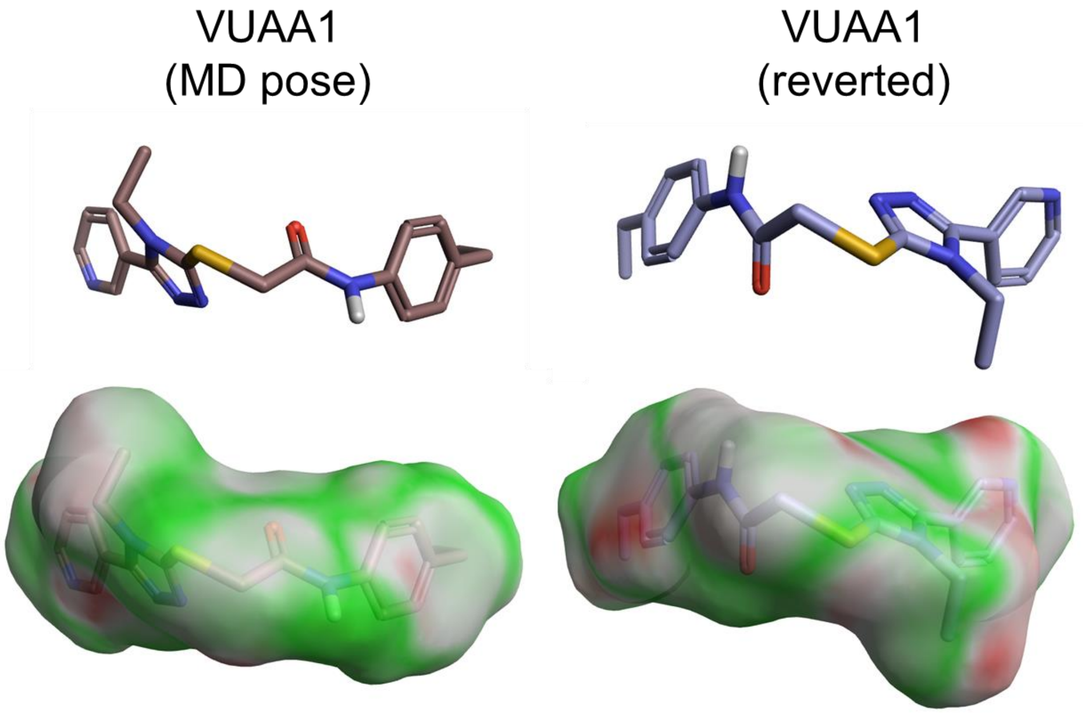

Supplementary Fig. 5 | Comparison of the orientation of VUAA1 in the binding site obtained during the MD simulations (MD pose) and the manually reversed orientation. The electrostatic complementarity of the ligand to the protein appears as a surface. The areas where the protein-ligand electrostatics are favorable or unfavorable are colored from green to red.

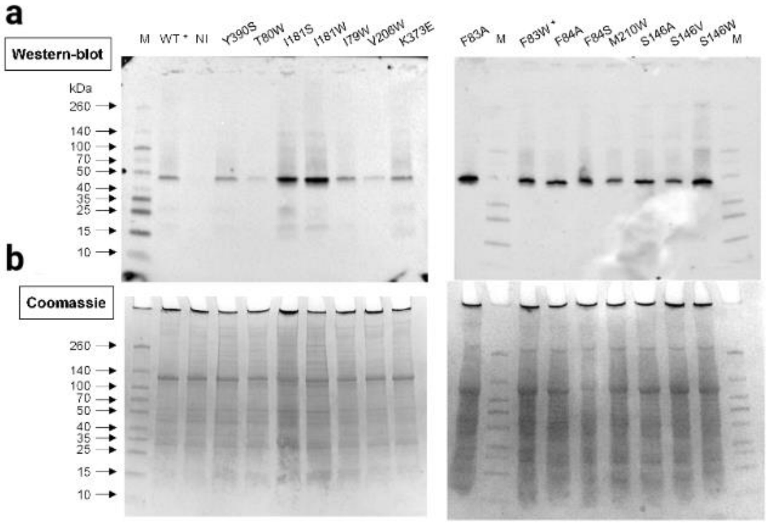

c Relative intensities

	Western-blot	Coomassie	WB/Coomassie
WT *	1.0	1.0	100%
NI		1.3	
Y390S	1.0	1.0	109%
T80W	0.3	1.5	18%
I181S	5.3	2.5	211%
I181W	6.6	2.2	296%
I79W	1.2	1.5	78%
V206W	0.3	1.1	30%
K373E	1.2	1.4	85%
F83A	1.5	1.0	158%
F83W *	1.0	1.0	100%
F84A	1.0	0.8	131%
F84S	0.8	0.7	118%
M210W	0.6	0.8	72%
S146A	0.9	0.8	108%
S146V	0.9	0.8	108%
S146W	1.3	1.0	134%

Supplementary Fig. 6 | Western-blot analysis of DmelOrco WT and mutant expression in *Xenopus* oocytes. **(a)** Western blots with a polyclonal primary antibody directed against DmelOrco. The constructs indicated above the lanes were expressed in *Xenopus* oocytes and the samples are crude membrane extracts. M: ThermoScientific Spectra Multicolor Broad Range Protein Ladder; WT*: wild-type used as reference for determining the relative intensities of bands of the first blot and gel, while the mutant F83W* was used for the second blot and gel. **(b)** Coomassie blue –stained SDS PAGE gels (4-20%). **(c)** Values of relative intensities of the bands corresponding to Orco (Western-blot column) in panel A and to the lanes (Coomassie) in panel B. Corrected intensities of bands relative to the intensities of lanes are indicated in the column WB/Coomassie.

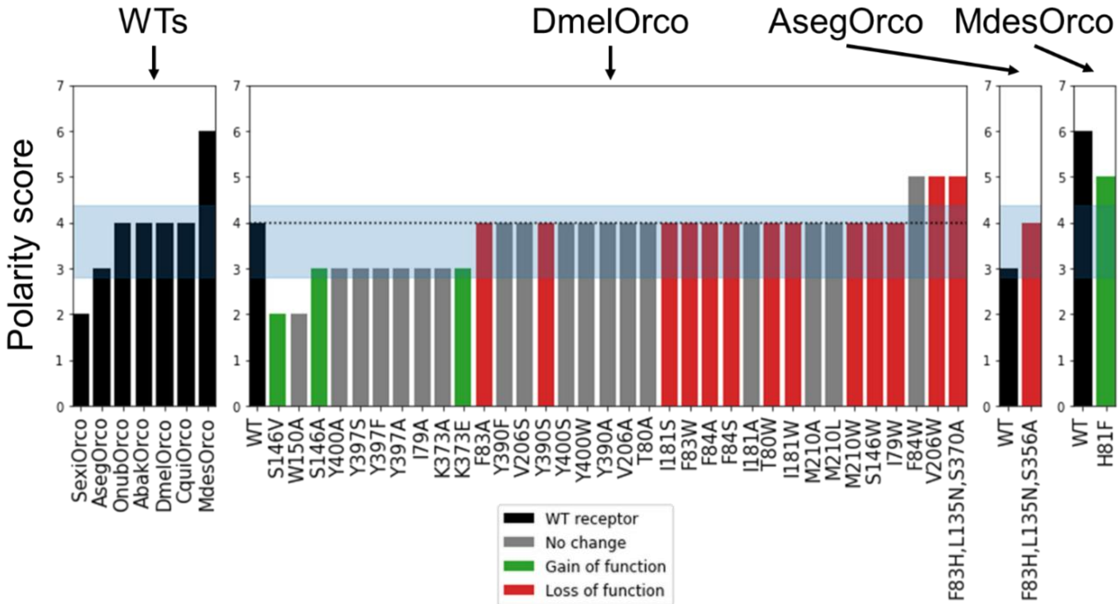

Supplementary Fig. 7 | Evolution of the polarity score of DmelOrco's mutants and from other species computed with fpocket. On the left, the results for WT Orco; for clarity, only the VUAA1-responsive and VUAA1-non-responsive Orcos are shown. SexiOrco and MdesOrco are the receptors with the lowest and highest scores, respectively. The central panel gathers the results for DmelOrco mutants. The two right panel gathers results for AsegOrco and MdesOrco. The blue bands account for the mean and standard deviation among 176 Orcos from 174 species.

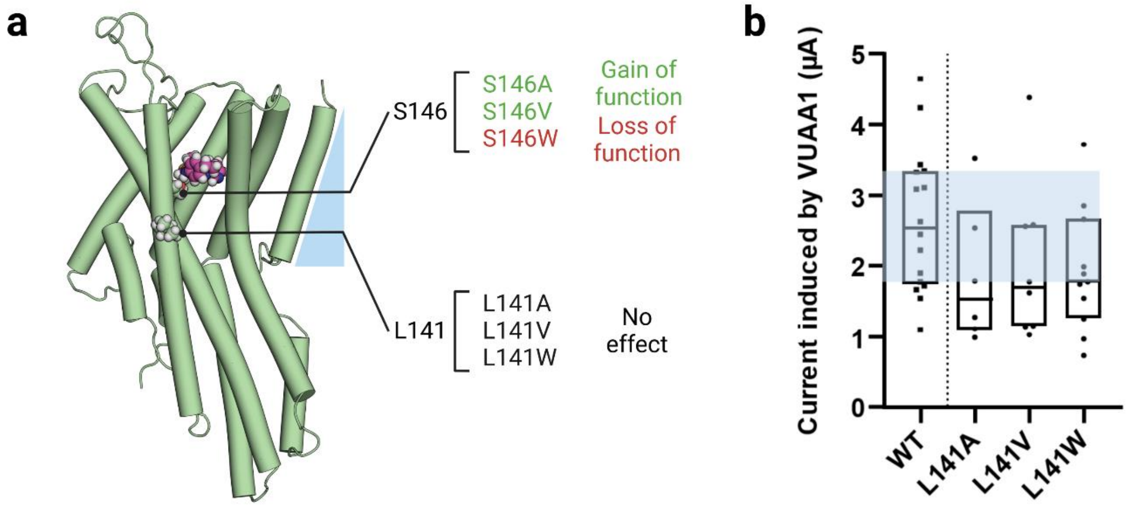

Supplementary Fig. 8 | Characterization of mutations at the position L141 that is not involved in the binding site nor in the translocation pathway of VUAA1. (a) Location of the L141 in the model of DmelOrco. For comparison, S146 was also depicted. The carbon atoms from the residue are in pale green, while the ones of VUAA1 are in purple. **(b)** Boxplot showing the current induced by 500 µM of the ligand VUAA1 and measured by TEVC recordings on DmelOrco WT and mutants expressed in *Xenopus* oocytes. The lower limit of the box plot represents the first quartile, while the upper limit represents the third quartile. Center bar correspond to the median. The colored blue area is a projection of the WT box plot. n=16 (WT), n= 6 (L141A), n=8 (L141V), n=11 (L141W). P values are 0.290, 0.396, 0.192 for L141A, L141V and L141W, respectively. Data are analysed with one-way ANOVA with α-error= 0.05 followed by Dunn's post-hoc test, with WT used as reference for multiple comparisons test.

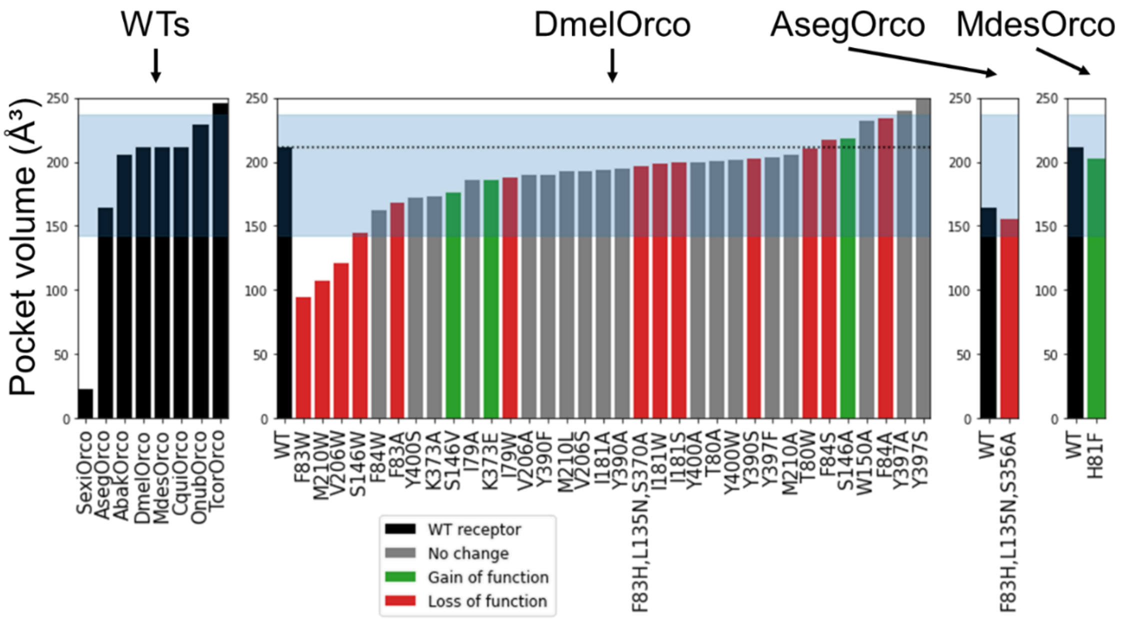

Supplementary Fig. 9 | Evolution of the binding cavity volume of DmelOrco's mutants and from other species computed with fpocket. On the left, the results for WT Orcos. For more clarity, only the VUAA1-responsive and VUAA1-non-responsive Orco are shown. SexiOrco and TcorOrco are the receptors with the lowest and highest volume, respectively. The central panel gathers the results for DmelOrco mutants. The two right panel gathers results for AsegOrco and MdesOrco. The blue bands account for the mean and standard deviation among 176 Orcos from 174 species.

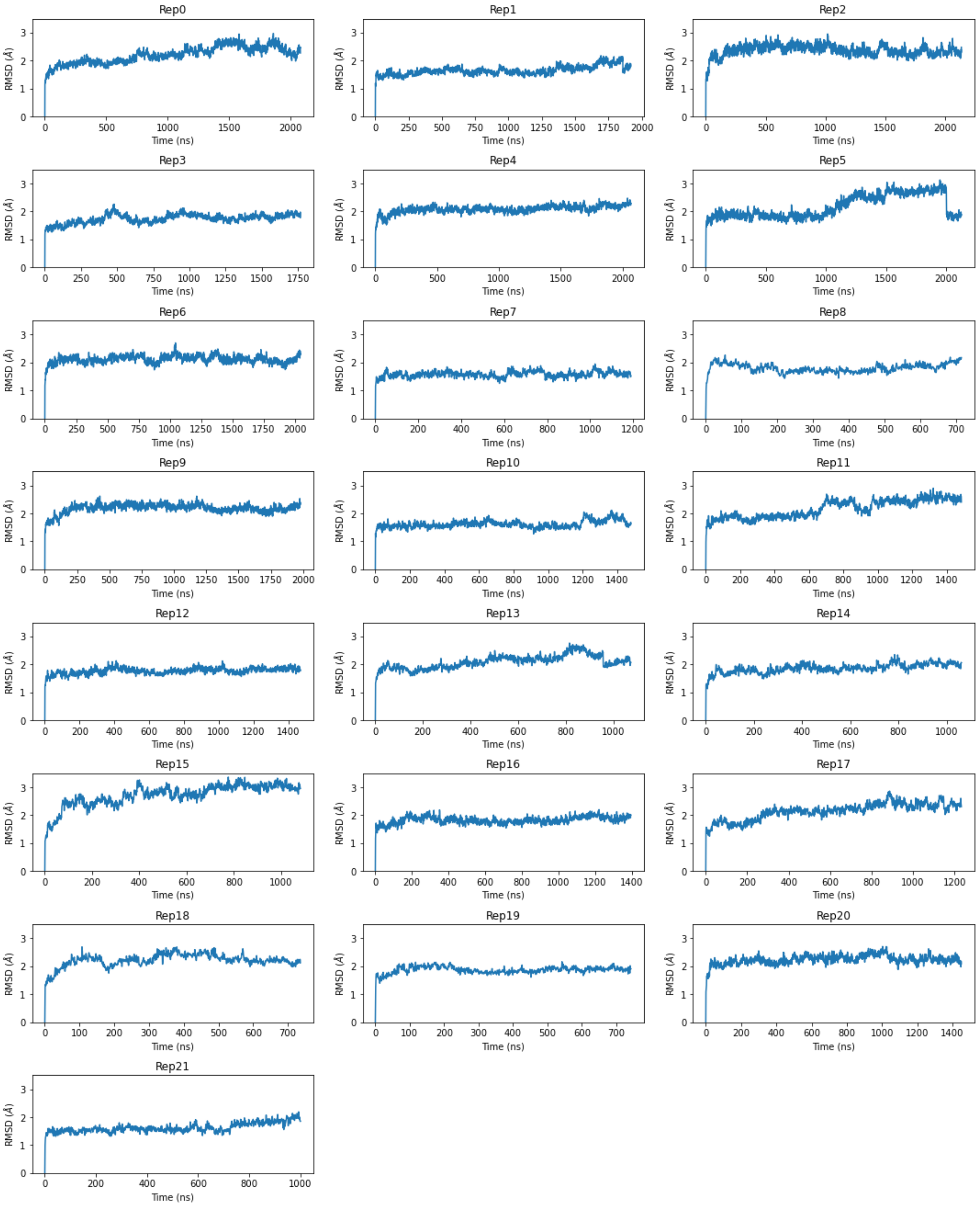

Supplementary Fig. 10 | Stability of the DmelOrco model in 22 replicas. The RMSD is computed for the receptor backbone (CA, C, N atoms) with respect to the initial model structure.

a)

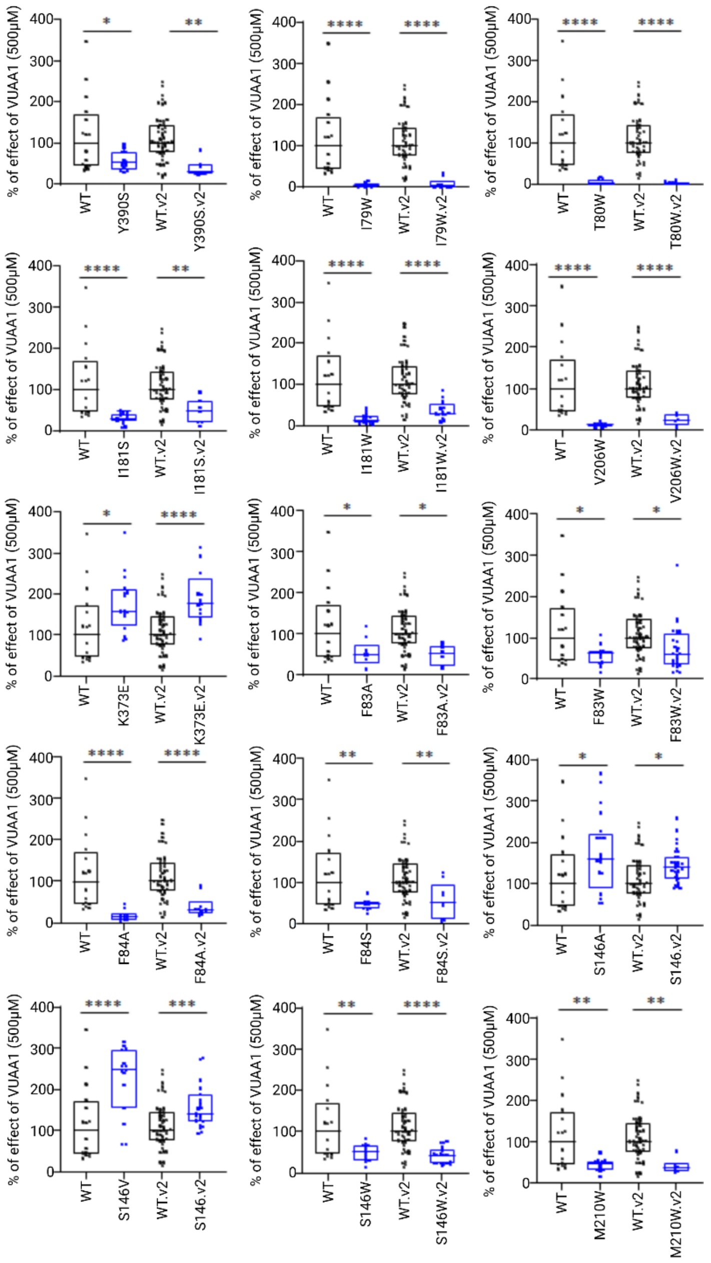

b)

WT or mutant DmelOrco	WT	Y390S	I79W	T80W	I181S	I181W	V206W	K373E	F83A	F83W	F84A	F84S	S146A	S146V	S146W	M210W
median current (µA)	4.71	2.44	0.14	0.03	1.40	0.54	0.59	7.47	2.33	2.94	0.68	2.19	7.55	11.68	2.44	2.12
% of activation	100.0	51.9	3.0	0.6	29.8	11.5	12.5	158.7	49.5	62.4	14.4	46.6	160.5	248.3	51.9	45.0

WT or mutant DmelOrco v2	WT.v2	Y390S.v2	I79W.v2	T80W.v2	I181S.v2	I181W.v2	V206W.v2	K373E.v2	F83A.v2	F83W.v2	F84A.v2	F84S.v2	S146A.v2	S146V.v2	S146W.v2	M210W.v2
median current (µA)	3.37	0.97	0.07	0.03	1.59	0.97	0.77	5.95	1.74	2.06	1.07	1.70	4.70	4.74	1.45	1.22
% of activation	100.0	28.8	2.1	0.9	47.2	28.8	22.9	176.6	51.6	61.1	31.6	50.5	139.5	140.7	42.9	36.1

Supplementary Fig. 11 | Second set of experiments. a) Relative responses to 500 µM VUAA1 of mutants (blue bars) compared to WT (black bars) in 2 different sets of experiments. Percentages are calculated based on the median of WT current amplitudes as 100%. The experiments in the 2nd set (noted –v2) have been performed in the same day and with the same batch of oocytes as WT-v2. n=49 (WT.v2), n=7 (Y390S.v2; I181S.v2), n=9 (I79W.v2; F84S.v2), n=14 (T80W.v2),n=15 (I181W.v2), n=6 (V206W.v2; F83A.v2; M210W.v2), n=16 (K373E.v2), n=24 (F83W.v2), n=10 (F84A.v2), n=31 (S146A.v2), n=23 (S146V.v2), n=20 (S146W.v2) recordings from different oocytes. P values are symbolized over bars as black stars, each star being one decimal. Data are analysed with one-way ANOVA with α-error= 0.05 followed by Dunn's post-hoc test, with WT used as reference for multiple comparisons test. ****p<0.0001, **p=0.0010 (Y390S.v2), *p=0.0145

(F83A.v2), *p=0.0218 (F83W.v2), **p=0.0071 (F84S.v2), *p=0.0165 (S146A.v2), ***p=0.0007 (S146V.v2), **p=0.0032 (M210W.v2). b) Table gathering the median current and the percentage of activation relatively to the WT.

Supplementary Table 1 | Electrostatic and hydrophobic complementary between the series of VUAA1 analogues and different stable locations in the protein. The locations are defined according to Figure 3: entry (b), vestibule (c) and binding site (d). The analogues have been ranked according to the EC_{50} measured by Tailor *et al*. on AgamOrco/AgamOR65[1]. The reversed ligand accounts for the reversed orientation described in Supplementary Fig. 9.

Location	Ligand (EC_{50})	Hydrophobic complementarity	Electrostatic complementarity
Entry (b)	VUAA0.5	0.37	0.26
	VUAA1	0.41	0.29
	VUAA2	0.44	0.31
	VUAA3	0.46	0.33
	VUAA4	0.45	0.32
Vestibule (c)	VUAA0.5	0.61	0.28
	VUAA1	0.62	0.30
	VUAA2	0.63	0.31
	VUAA3	0.64	0.29
	VUAA4	0.66	0.29
Binding site (d)	VUAA0.5 (110 μM)	0.65	0.33
	VUAA1 (37 μM)	0.64	0.34
	VUAA2 (9.2 μM)	0.64	0.36
	VUAA3 (8.4 μM)	0.71	0.36
	VUAA4 (2.1 μM)	0.72	0.36
Binding site (d) with reversed ligand	VUAA0.5	0.58	0.28
	VUAA1	0.58	0.31
	VUAA2	0.58	0.31
	VUAA3	0.65	0.30
	VUAA4	0.64	0.32

The electrostatic complementarity of the VUAA series in the binding site show little differences in EC values, although it shows a correct trend (Pearson correlation of -0.93). These small differences are in the same order of magnitude with a previous study from Bauer *et al.*[2] In their study, the 6 ligands are discriminated based on an EC score with subtle EC variations (lig18: IC_{50} = 2nM, EC = 0.425; lig23: IC_{50} = 447nM, EC = 0.385).

Supplementary Table 2 | EC_{50} and Imax of VUAA1 from WT and mutated DmelOrco. ND means Not Determined due to the absence of plateau. Separation lines differentiate WT from mutants in the vestibule (Figure 4) and the binding site (Figure 5), in this respective order. Within separation lines, mutants are ranked in descending order of Imax, which is measured in concentration-effect curves and slightly lower than the Imax measured in single-concentration experiments.

	EC_{50} (μM)	I_{max} (μA)
**WT**	94.5	3.97
**I181S**	101.9	0.94
**I79W**	ND	0.65
**V206W**	ND	0.33
**T80W**	ND	0.23
**S146V**	82.2	6.99
**M210W**	120.2	1.50
**F84A**	105.0	1.01

Supplementary Table 3 | Comparison of the mutation effects on MhraOR5, MhraOR1 eugenol-induced channel response and DmelOrco VUAA1-induced channel response. A, D, I, na and nt mean Abolished, Decreased, Increased, non-affected and not tested, respectively. Results for MhraOR5 and MhraOR1 are taken from Del Marmol et al.[3] Each line corresponds to the same position in the three considered proteins MhraOR5, MhraOR1 and DmelOrco. Alignment between the sequences was taken from ref[3].

	MhraOR5	Effect on Eugenol	Effect on DEET	MhraOR1	Effect on Eugenol	DmelOrco	Effect on VUAA1
Ligand diffusion	T87A	D		L102A	na	I79A	na
	L379A	na		L398A	na	G399	nt
Binding site	V88A	D		T103	A	T80A	na
	Y91A	D		Y106A	A	F83A	D
	F92A	A		I107A	A	F84A	D
	S151A	A		S166A	A	S146A	I
	G154A	A		G169A	I	A149	nt
	W158A	A		W173A	A	W150A	na
	M209A	A	D	L227A	D	V206A	na
	M209V	D	I			V206S	n.a
	M209L	D	D			V206W	A
	I213A	A	A	M231A	A	M210A	na
	I231M	D	A			M210W	D
	L379A	na		L398A	na	G399	nt
	Y380A	A		Y339A	A	Y400A	na
	Y383A	A		C402A	na	A403	nt

Dataset S1 (Dataset S1.xlx). Sequence alignment of different Orco. The known mutations are indicated in color on the sequence.

Dataset S2 (Dataset S2.xlx). Contact frequencies between VUAA1 and amino-acids from Orco during the MD simulations. The first sheet gathers the contact frequencies for the simulations in which the ligand sampled the binding site. The second sheet gather the frequency when the ligand visits areas b, c or d but do not reach the binding site.

SI References

1. R. W. Taylor *et al.*, Structure–activity relationship of a broad-spectrum insect odorant receptor agonist. *ACS Chem. Biol.* **7**, 1647-1652 (2012).

2. M. R. Bauer, M. D. Mackey, Electrostatic complementarity as a fast and effective tool to optimize binding and selectivity of protein–ligand complexes. *J. Med. Chem.* **62**, 3036-3050 (2019).

3. J. Del Marmol, M. A. Yedlin, V. Ruta, The structural basis of odorant recognition in insect olfactory receptors. *Nature* **597**, 126-131 (2021).

Uncropped scans of blots and gels of the Supplementary Fig. 6. The primary antibody in western-blots is a polyclonal antibody directed against DmelOrco and made by order by Genscript. The molecular weight marker is the ThermoScientific Spectra Multicolor Broad Range Protein Ladder.

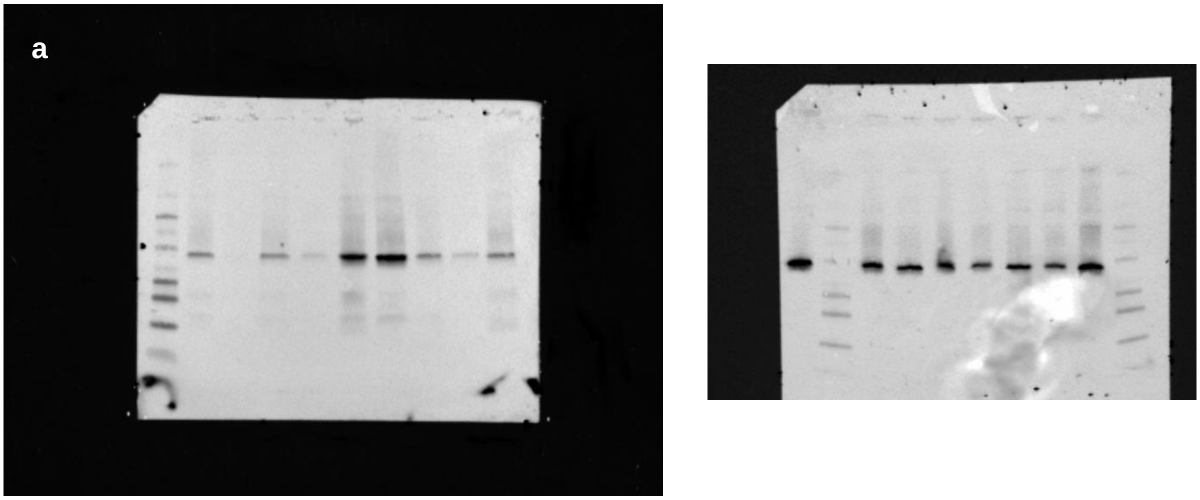

b

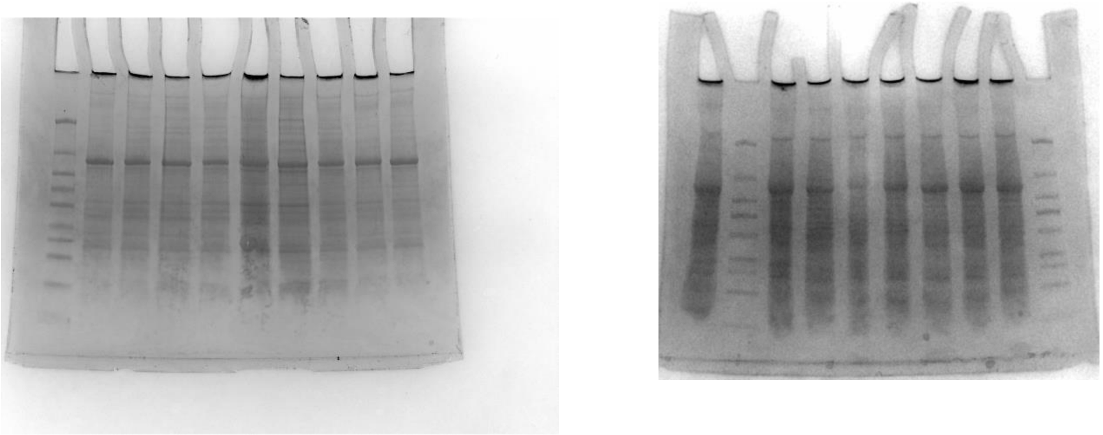

Supplement: Supplementary file 1 — Supplementary Information [file 41467_2023_44058_MOESM1_ESM.pdf]
