# Supplementary material for: Elucidation of the structural basis for ligand binding and translocation in conserved insect odorant receptor co-receptors
Source: Nat Commun. 2023 Dec 11;14:8182. doi: 10.1038/s41467-023-44058-5 (PMC10713630; doi:10.1038/s41467-023-44058-5)
Supplement: Supplementary file 3 — Description of Additional Supplementary Files [file 41467_2023_44058_MOESM3_ESM.pdf]

Description of Additional Supplementary Files

File Name: Supplementary Data 1
Description: Sequence alignment of different Orco. The known mutations are indicated in color on the sequence.

File Name: Supplementary Data 2
Description: Contact frequencies between VUAA1 and amino-acids from Orco during the MD simulations. The first sheet gathers the contact frequencies for the simulations in which the ligand sampled the binding site. The second sheet gather the frequency when the ligand visits areas b, c or d but do not reach the binding site.
